# LINC00473 promotes the Taxol resistance via miR-15a in colorectal cancer

**DOI:** 10.1042/BSR20180790

**Published:** 2018-09-21

**Authors:** Lin Wang, Xufeng Zhang, Li Sheng, Chun Qiu, Rongcheng Luo

**Affiliations:** 1 Cancer center, Integrated Hospital of Traditional Chinese Medicine, Southern Medical University, Guangzhou, China; 2 Department of Medical Oncology, Hainan General Hospital, Haikou, China; 3 Cancer center, the First Affiliated Hospital of Hainan Medical College, Hainan Medical College Cancer Institute, Haikou, China

**Keywords:** apoptosis, colorectal cancer, chemotherapy, LINC00473, miR-15a

## Abstract

Dysregulation of long non-coding RNAs (LncRNAs) participated into the initiation and progression of different diseases via direct regulation of proteins or indirect regulation of microRNA (miRNA)-target genes. LINC00473 is a novel carcinoma-related LncRNA and up-regulated in many cancers for tumor growth and metastasis, but its role in chemotherapy resistance is unclear. We here investigated the function of LINC00473 in colorectal cancer (CRC) *in vitro* and *in vivo*. The CRC tissues (*n*=20) and relative normal tissues were collected and found that LINC00473 was overexpressed in CRC tissues when compared with which in normal tissues. Highly expressed LINC00473 predicted large tumor size, high TNM stage of CRC patients. Interestingly, the tumor suppressor miR-15a was down-regulated and negatively correlated with LINC00473 levels in CRC. LINC00473 harbored the binding sites for miR-15a and reduced its availability in CRC cell line HCT116. Knockdown of LINC00473 elevated the expression of miR-15a. Moreover, in the Taxol-resistant HCT116, the LINC00473 level was further increased than that in HCT116. Knockdown of LINC00473 restored the Taxol-induced cytotoxicity, inhibited the cell vitality, colony formation and induced apoptosis, impaired the ability of migration or invasion, but these effects could be abrogated by the inhibition of miR-15a. Mechanistically, the BCL-2-related anti-apoptosis pathway was activated and the multidrug-resistant (MDR) genes LRP, MDR1 were up-regulated by LINC00473. Furthermore, inhibition of LINC00473 *in vivo* could overcome the Taxol resistance of CRC cells, could recover the expression of tumor suppressor miR-15a and chemotherapy-induced tumor regression, indicating that LINC00473 functioned as oncogene in CRC via miR-15a.

## Introduction

Colorectal cancer (CRC) is the third most prevalent malignancy worldwide with 130,000 estimated new cases diagnosed each year. It is also the fourth cause of cancer-related deaths with 69,000 estimated deaths in 2012 [1]. CRC consists of colon and rectal cancers and over 70 percent of cases arise in the colon. Once metastases occur in lymph nodes or distant sites, the overall 5-year survival is ~30% [2]. The high mortality rate primarily attribute to the lack of markers diagnosing CRC at early stages and of efficient alternative strategy of therapy [3]. Thus, understanding the exact etiology and pathology of CRC is conducive to identifying new diagnostic and prognostic biomarker and developing new therapy for CRC.

Mounting evidences have uncovered the critical role of non-coding RNAs (ncRNAs) in many physiological and pathological processes including carcinogenesis [4,5]. Due to the lack of protein-coding sequences, ncRNAs are the transcripts without coding potential. They are eventually processed into a heterogeneous group of molecules with different size and function, such as microRNAs (miRNAs) with 19–24 nucleotides, long non-coding RNAs (lncRNAs) with longer than 200 base pairs [6]. Aberrantly expressed miRNAs and LncRNAs have been proposed as prognostic or diagnostic biomarkers for cancers, including CRC [7–9]. The tumor suppressors, miR-34b and miR-34c, were down-regulated in advanced CRC tissues and decreased expression of miR-34b/c was associated with poor cancer-specific mortality [10]. The oncogene lncRNA HULC had been found to be up-regulated in gastric cancer and hepatocellular carcinoma, and was also overexpressed in CRC, which was associated with poor prognosis and shorter survival [11]. LINC00473 was a novel lncRNA that participated into tumor proliferation, chemotherapy resistance and human decidualization via cAMP/CREB signals [12,13]. Chen et al. [14] reported that elevated LINC00473 expression correlated with poor prognosis of patients with lung cancer, and sustained LINC00473 expression promoted CREB-mediated transcription to support the growth and survival of LKB1-inactivated lung cancer cells. However, the function of LINC00473 in CRC is poorly understood.

Interactions with miRNAs and proteins are critical mechanisms for LncRNAs to exert their function. LncRNAs could inhibit the availability of miRNAs or be targeted and inhibited by miRNA [15]. Vertebrate lncRNA H19 harbors both canonical and non-canonical binding sites for the miRNA let-7 family and could modulate let-7 availability by acting as a molecular sponge [16]. The interaction between LINC00473 and miRNA has been found. Shi et al. [17] found that miR-34a targeted and reduced the stability of LINC00473 in cervical cancer, leading to the degradation of oncogene interleukin enhancer binding factor 2 (ILF2) and tumor inhibition. LINC00473 interacts with the transcript factor C/EBPβ to facilitate its binding to the promoter of IL24, leading to recover the cisplatin-induced apoptosis in osteosarcoma [18]. Currently, the function of LINC00473 in chemotherapy resistance is still limited. In the present study, we focused on the clinical role of LINC00473 in CRC patients and also investigated its role in Taxol-dependent chemotherapy *in vitro* and *in vivo*.

## Materials and methods

### Tumor samples

To determine the expressions of LINC00473 and miR-15a in CRC patients, the CRC tissues (*n* = 20) and relative normal tissues were collected after obtaining informed consents according to procedures approved by the Ethics Committee at Hospital of Traditional Chinese Medicine, Southern Medical University. The tissue samples were isolated from surgical removal and then stored at −80°C until use.

### Cell lines and reagents

The normal human colon epithelial cell line FHC and four CRC cell lines HCT116 and Taxol-resistant CRC cell line HCT116/ Taxol, SW620 and LoVo were obtained from the cell bank of the Chinese academy of sciences (Shanghai, China) and cultured in Dulbecco’s Modified Eagle’s medium (DMEM) supplemented with 10% FBS (Life Technologies, U.S.A.), ampicillin and streptomycin at 37°C, 5% CO_2_ conditions. Anti-GAPDH, BCL-2, caspase-3, BAX, LRP, MDR1 and Ki-67 antibodies were obtained from Cell Signaling Tech (Denver, MA) and Abcam (U.S.A.). siRNA-LINC00473 or negative control was purchased from RiboBio (Guangzhou, China). Renilla luciferase reporter vector psiCHECK2- LINC00473-wild or mut were conducted by GenePharma (Shanghai, China). The oligonucleotide sequences of miR-15a mimics, inhibitors or negative control were purchased from RiboBio (Guangzhou, China). Five- to six-week-old male BALB/c-nu/nu nude mice were purchased from Experimental animal center of Southern Medical University (Changzhou, china).

### RNA isolation and qRT-PCR

To analyze the expression of LINC00473 and miR-15a in tumor tissues and cell lines, total RNA from tissues or tumor cells was extracted using Trizol reagent (Invitrogen) according to the standard RNA isolation protocol. Quantitative real-time RT-PCR (qRT-PCR) was performed, and 2^−ΔΔ*C*^_t_ method was used to estimate relative expressions of LINC00473 and miR-15a, which were normalized to GAPDH and U6 for gene expression.

### Cell transfection

After the HCT116/Taxol cells were cultured to ~80% confluence, siRNA-LINC00473 or psiCHECK2- LINC00473-wild or mut and negative control were transfected into HCT116 cells using Lipofectamine 2000 (Invitrogen, U.S.A.) according to the manufacturer’s instructions. After transfection for the indicated time, the cells were harvested for further experiments.

### Luciferase reporter assay

The HCT116/Taxol cells were co-transfected containing psiCHECK2-LINC00473-wild or mut and miR-15a mimics. Luciferase activity was measured using the Dual-Luciferase Reporter Assay System (Promega, U.S.A.). Firefly luciferase acted as a reporter gene for normalized control.

### CCK-8 assay

The HCT116/Taxol cells were transfected with negative control, siRNA- LINC00473 and/or miR-15a inhibitor for 24, 48 and 72 h. The cells were harvested and washed with PBS and then cell counting kit-8 (Kumamoto, Japan) mixed with DMEM was used for cell viability assay, and the absorbance was measured at 450 nm by a microplate reader.

### Colony formation assay

The HCT116/Taxol cells were transfected with negative control, siRNA- LINC00473 and/or miR-15a inhibitor. Then the conditional cells were harvested and were re-suspended in complete medium containing 10% FBS and were seeded into 12-well plates for 10 days. The cells fixed with methanol for 15 min were visualized by 0.1% Crystal Violet under a dissection microscope (Olympus, Japan) and colonies consisting of 50 cells or more were counted.

### Determine of apoptosis

The apoptosis of HCT116/Taxol cells were determined by flow cytometry. Briefly, after the collection of cells by suspending, 2 μl of annexin V mixed with 2 μl of propidium iodide (PI, eBioscience) were used to stain dispersed cells, 10,000 cells were collected for the analysis of flow cytometry. The cell cycle distribution was analyzed using a flow cytometry provided with the Cell-Quest software.

### Transwell assay

About 3 × 10^4^ HCT116/Taxol cells transfected with negative control, siRNA-LINC00473 and/or miR-15a inhibitor were placed in the upper chamber of a non-coated transwell insert for migration assay and of a transwell insert coated with Matrigel for invasion assay. After migration for 12 h, cells that did not migrate or invade were removed using a cotton swab and were stained by 0.1% Crystal Violet and counted under an inverted microscope. Five random views were selected to count the cells.

### Western blots

The whole cell protein extracts from cell lines or tumor tissues were prepared according to the manufacturer’s instructions, and were separated by a 10% SDS denatured polyacrylamide gel. Proteins were then transferred to a polyvinylidene difluoride membrane (Millipore, Bed-ford, MA). Filters were blocked overnight in 5% BSA and incubated with primary antibodies anti-GAPDH, BCL-2, caspase-3, BAX, LRP and MDR1 overnight at 4°C. After washing with TBST buffer for three times, the blots were then incubated with HRP-conjugated secondary antibody for 2 h at room temperature. After washing with TBST buffer, the blots were visualized using the ECL-Plus reagent (Millipore, Billerica, MA, U.S.A.). GAPDH was used as the loading control in the Western blotting.

### Tumor model

The xenograft model of human HCT116/Taxol cells was established. The HCT116/Taxol cells were transfected with lentivirus vector of siRNA-LINC00473 or negative control, 2 × 10^6^ tumor cells were subcutaneously injected in rear flank of nude mice (six per group). The mice were treated with 6 mg/kg Taxol (i.p) three days apart for six times. The tumor sizes were measured three days apart and the tumor volumes were calculated: *V* (cm^3^) = [width^2^ (cm^2^) * length (cm)] / 2. After the termination of Taxol administration, the mice were killed and the tumor weights were also determined.

### Immunohistochemistry

The expressions of Ki-67 in CRC tissues were analyzed by IHC as previously described [19]. The EnVision Detection System kit (DAKO, Denmark) was used for the DAB chromogen followed by nuclear staining using hematoxylin.

### Statistical analysis

The statistical analyses were performed by GraphPad Prism 5.0 (GraphPad Software, La Jolla, CA, U.S.A.). All data were expressed as mean ± SD. Unpaired *t*-tests or Mann–Whitney *U* tests were used to compare the two groups, and multiple group comparisons were analyzed with one-way ANOVA. *P*<0.05 was considered to be statistically significant.

## Results

### Up-regulated LINC00473 positively correlated with poor clinical outcome of CRC patients

**Figure 1:**
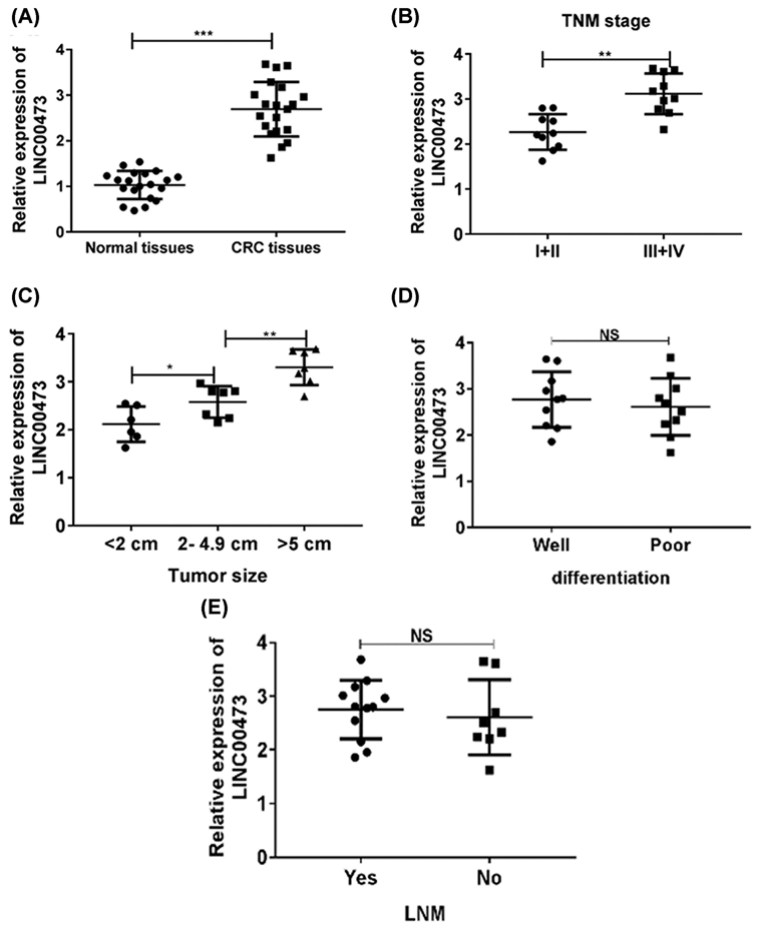
LINC00473 is overexpressed in CRC patients (**A**) The expression of LINC00473 was analyzed in CRC tissues and relatively normal tissues (*n*=20) were by Q-PCR. (**B**–**E**) Correlations between the expression of LINC00473 and clinicopathological characteristics (tumor size, TNM stage, differentiation and LNM status) of CRC patients were analyzed. *P<0.05, **P<0.01, ***P<0.001; data represent the means ± s.d.

We first investigated the clinical significance of LINC00473 in CRC samples. Colon cancer tissues (*n*=20) and relative normal tissues (*n*=20) were collected. The results from Q-PCR indicated that the expression of LINC00473 was increased in tumor tissues than that in relative normal tissues (Figure 1A). We next analyzed the correlation between the clinicopathological characteristics of CRC patients and LINC00473 expression. The results indicated that higher expression of LINC00473 was positively associated with larger tumor size and higher TNM stage (Figure 1B,C). However, no correlations were observed between cell differentiation, lymph node metastasis and LINC00473 expression (Figure 1D,E). These data indicated that highly expressed LINC00473 might participate into the tumor progression of CRC.

### Tumor suppressor miR-15a is targeted and inhibited by LINC00473

**Figure 2:**
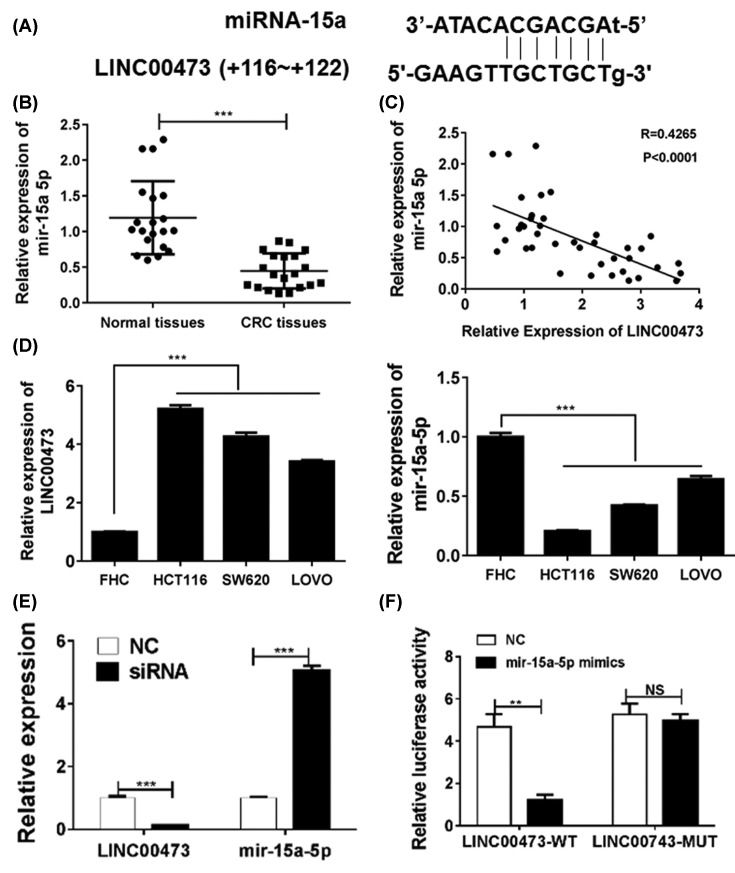
LINC00473 interacts with miR-15a and inhibits its expression in CRC (**A**) The predicted miR-15a-binding sites for LINC00473. (**B**) The expression of miR-15a was analyzed in CRC tissues and relatively normal tissues were by Q-PCR. (**C**) The correlation between miR-15a and LINC00473 in CRC tissues was determined. (**D**) The expressions of miR-15a and LINC00473 were confirmed in normal human colon epithelial cell line FHC and four CRC cell lines HCT116, SW620 and LoVo. (**E**) Knockdown of LINC00473 could increase the miR-15a levels in HCT116 cell line. (**F**) The HCT116 cell line was transfected with miR-15a mimics, psiCHECK2-LINC00473-wild or mut and negative control, and then the luciferase activity was measured. **P<0.01, ***P<0.001, data represent the means ± s.d.

To investigate the mechanism of LINC00473-related carcinogenesis, we determined the expression and function of LINC00473 in CRC cell lines *in vitro*. The interactions between LncRNAs and miRNAs have been found to be the important way to regulated miRNA-targeting genes. Bioinformatic analysis revealed putative complementary sequences for miR-15a in human LINC00473 and a predicted tumor suppressor miR-15a-binding sites were found (http://starbase.sysu.edu.cn/index.php) (Figure 2A). The expressions of miR-15 in CRC tissues were analyzed and found that the miR-15a levels were significantly reduced in tumor tissues, which was negatively correlated to the LINC00473 level (Figure 2B,C). In addition, the expression of LINC00473 and miR-15a were also confirmed in normal human colon epithelial cell line FHC and four CRC cell lines HCT116, SW620 and LoVo. The results showed that the CRC cell lines had higher expressions of LINC00473 but lower miR-15a levels when compared with the FHC cell lines (Figure 2D).

Knockdown of LINC00473 in HCT116 cell lines could significantly up-regulated the levels of miR-15a (Figure 2E). To provide the direct evidence, a luciferase reporter vector with full-length (wild-type or mutant miR-15a-binding sites) of LINC00473 was transfected into HCT116 cell line to perform the luciferase reporter assays and the results showed that miR-15b mimics could significantly impair the luciferase activity of wild-type LINC00473, which was not observed in the mutant-type, indicating that LINC00473 direct interacted with miR-15a and inhibited the expression in CRC cells (Figure 2F).

### LINC00473 promotes the Taxol resistance via miR-15a inhibition

**Figure 3:**
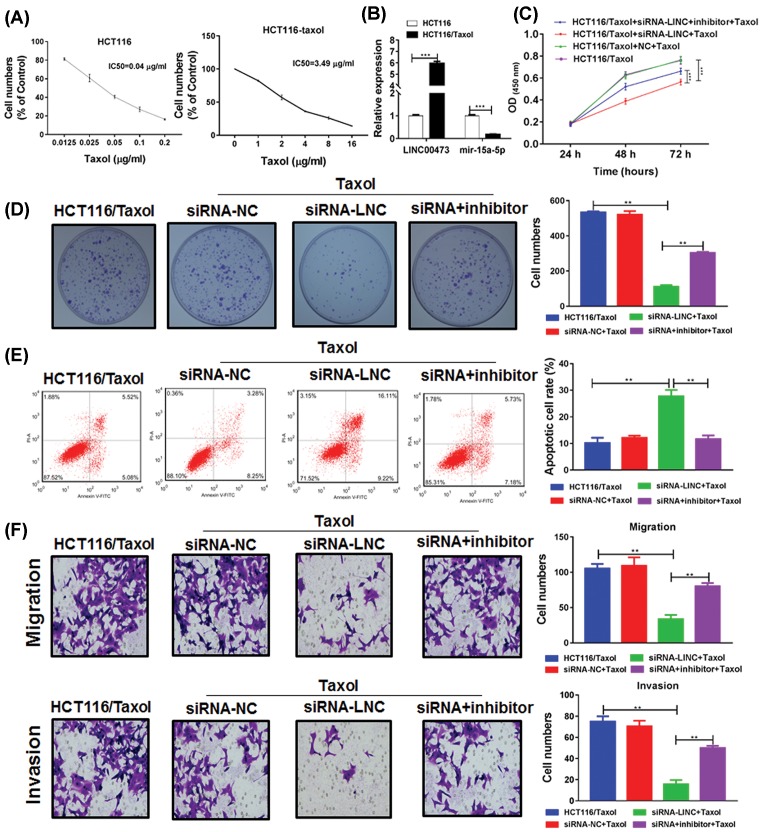
LINC00473 promotes Taxol resistance via miR-15a in CRC cells (**A**) The IC50 of Taxl in HCT116/Taxol and HCT116 was determined by CCK-8. (**B**) The expressions of miR-15a and LINC00473 in HCT116/Taxol and HCT116 cells were determined by Q-PCR. (**C**) The HCT116/Taxol cells were transfected of siRNA of LINC00473 with or without miR-15a inhibitors, then the cells were treated with 0.5 μg/ml Taxol and the cell vitality was estimated by CCK-8. Besides, (**D**) the proliferation, (**E**) apoptosis and (**F**) the ability of migration and invasion were determined by colony formation assay, flow cytometry and transwell assay. *P<0.05, **P<0.01, ***P<0.001, data represent the means ± s.d.

We next focused on its role in Taxol resistance. The Taxol-resistant CRC cell line HCT116/Taxol have higher IC50 than Taxol-non-resistant CRC cell line HCT116 (Figure 3A). Interestingly, compared with the HCT116, the HCT116/Taxol cell line had higher expression of LINC00473 but lower expression of miR-15a, indicating that LINC00473 could be associated with Taxol resistance (Figure 3B). After the treatment of Taxol, the HCT116/Taxol cells without LINC00473 knockdown showed no reduction in cell vitality, but the HCT116/Taxol cells with LINC00473 knockdown restored the sensitivity to Taxol-induced cytotoxicity. Meanwhile, the addition of miR-15a inhibitor could abrogate the LINC00473-restored chemotherapy sensitivity (Figure 3C). Moreover, the colony formation was also investigated and the results showed that inhibition of LINC00473 promoted Taxol-induced suppression of colony formation, which was impaired by miR-15a inhibitors (Figure 3D). The similar results were observed in apoptosis, migration and invasion assay (Figure 3E,F). Thus, knockdown of LINC00473 contributed to the recovery of sensitivity to Taxol in HCT116/Taxol cells via up-regulation of tumor suppressor miR-15a, leading to the inhibition of tumor growth and the ability of migration or invasion.

### LINC00473 impairs the apoptosis pathway and down-regulates MDR genes

**Figure 4:**
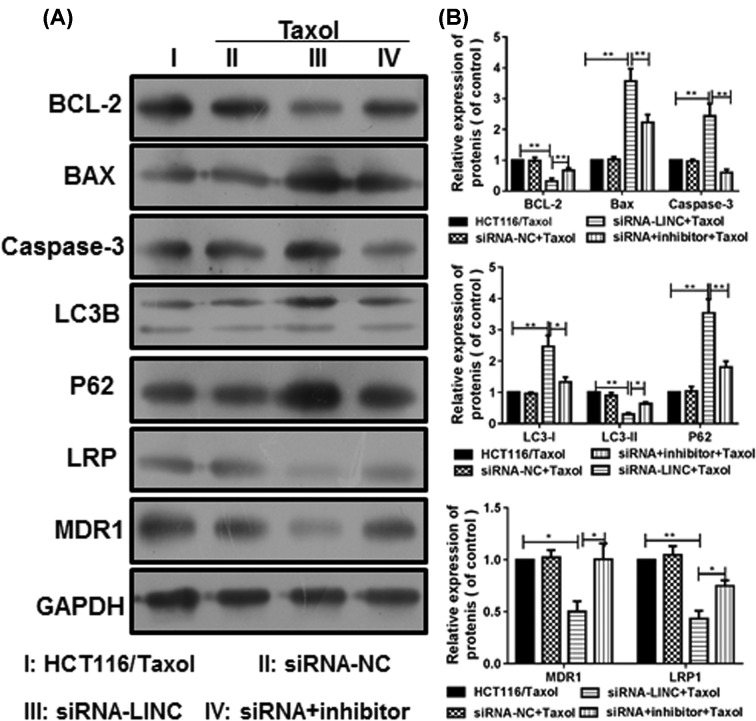
The apoptosis pathway and MDR genes were regulated by LINC00473 (**A**) The expressions of apoptosis-related Bcl-2, caspase-3, BAX and MDR-related LRP and MDR1 were determined by WB. (**B**) The grayscale value of each protein was analyzed. *P<0.05, **P<0.01, data represent the means ± s.d.

The underling molecular signals of LINC00473 knockdown were determined in HCT116/Taxol cells. The apoptosis pathway was assessed by WB (Figure 4A,B). The results indicated that the anti-apoptosis factor BCL-2 was down-regulated by LINC00473 knockdown, the expression of caspase-3 was also inhibited but the pro-apoptosis factor BAX was up-regulated. Thus, the apoptosis pathway was activated by LINC00473 knockdown. However, miR-15a inhibition impaired these effects and increased the BCL-2 level but decreased BAX level in HCT116/Taxol cells. Additionally, LINC00473 knockdown could also suppress the multidrug-resistant (MDR) genes LRP and MDR1 via miR-15a, which resulted in decreased resistance to Taxol and promoted apoptosis.

### Knockdown of LINC00473 overcomes the Taxol resistance *in vivo*

**Figure 5:**
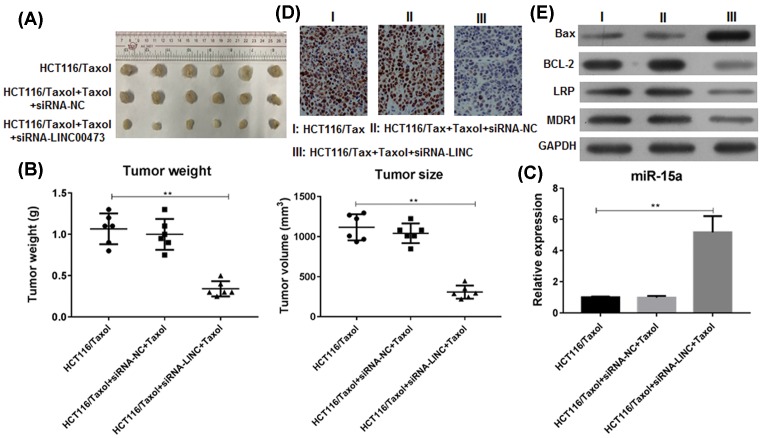
Knockdown of LINC00473 promotes the tumor regression of CRC *in vivo* HCT116/Taxol cells with/without LINC00473 knockdown were subcutaneously injected in rear flank of nude mice (six per group) and then the mice were treated with 6 mg/kg Taxol (i.p). (**A**) The mean tumor size (mm^3^) and (**B**) tumor weight (g) was analyzed. (**C**) The expression of miR-15a in tumor tissues was estimated by Q-PCR. (**D**) The expression of Ki-67 was determined by immunohistochemistry and (**E**) the expressions of apoptosis-related Bcl-2, caspase-3, BAX and MDR-related LRP and MDR1 in tumor tissues were determined by WB. **P<0.01, data represent the means ± s.d.

The xenograft model of human HCT116/Taxol cells was established. The nude mice were administrated of conditional tumor cells with/without LINC00473 knockdown and then were treated with Taxol. The results indicated that Taxol could not induced tumor regression of mice without LINC00473 knockdown, the mice with LINC00473 knockdown, however, efficiently responded to the treatment of Taxol and inhibited tumor growth (Figure 5A). The tumor weight was also significantly decreased by LINC00473 knockdown (Figure 5B). We further confirmed that this tumor inhibition was associated with the up-regulation of tumor suppressor miR-15a in tumor tissues (Figure 5C) and the down-regulation of proliferation index Ki-67 (Figure 5D). The apoptosis pathway was also estimated in tumor tissues. LINC00473 knockdown *in vivo* also inhibited the expression of BCL-2 but increased the expression of BAX and caspase-3, and suppressed the MDR genes LRP and MDR1 (Figure 5E). These data indicated that knockdown of LINC00473 *in vivo* recovered the availability of miR-15a and overcame the Taxol resistance in CRC.

## Discussion

The CRC patients with tumors arising in submucosa or muscularis propria have better prognosis with ~70% overall 5-year survival and can be treated successfully with surgical resection. However, over 50% of patients have developed to be advanced stage when diagnosed and they are refractory to existing therapies including chemotherapy and targeted biologic therapies [20]. NcRNAs have been identified to be involved into development, immunity and cancer. The ncRNAs targeting therapy also provide alternative efficient strategy for cancer treatment [21]. In the present study, we found that LINC00473 functioned as an oncogene in CRC, overexpressed LINC00473 inhibited Taxol-induced apoptosis and the suppression of CRC cell vitality, migration and invasion.

Currently, the expression profiles of ncRNAs have been found to be shifted during the carcinogenesis of CRC. MiR-21 has been described as one of the most highly overexpressed miRNAs in CRC, inhibits several genes involved in controlling invasion and migration, including PDCD4, TIAM1, SPRTY and PTEN [22]. MiR-144 was markedly down-regulated in colorectal cancer cells and suppressed the expression of GSPT1 to regulate the cell proliferation-related gene expressions of c-myc, survivin and Bcl2L15 and metastasis-related factor MMP28 [23]. MiR-204 is frequently down-regulated in colorectal cancer tissues, which was associated with poor prognoses [24]. We here reported that the tumor suppressor miR-15a was also down-regulated in CRC tissues when compared with normal tissues. Besides, many lncRNAs were also dysregulated in CRC tissues. AFAP1-AS1 was found to be overexpressed in CRC tissues and positively associated with TNM stage, tumor size and distant metastasis [25]. CRC-associated lncRNA (CCAL), also named as CCAT, was an oncogene to induce MDR through activating Wnt/β-catenin signals by suppressing AP-2α and further elevated MDR1/P-gp expression [26]. CRC patients with higher CCAT expression had a shorter overall survival and a worse response to adjuvant chemotherapy. We here found that LINC00473 was overexpressed in CRC tissues, which correlated with large tumor size and TNM stage, knockdown of LINC00473 inhibited the expressions of BCL-2, MRD gene and up-regulated the BAX for tumor killing, suggesting that LINC00473 was also an oncogene in the development of CRC.

Previous studies have demonstrated that LINC00473 was highly expressed in tumor for tumor progression, but its role in therapy resistance remains to be further elucidated. CRTC1–MAML2 fusion was a major oncogenic driver for mucoepidermoid carcinoma initiation and maintenance, CRTC1–MAML2 fusion remarkably up-regulated LINC00473 transcription to promote the binding to a cAMP signaling pathway component NONO and subsequently activate CREB-mediated transcription [12]. In fibrolamellar carcinoma (FLC), Dinh et al. [27] reported that LINC00473 up-regulated in FLC and was a unique lncRNA that distinguishes FLC from other liver and non-liver cancer types. In the present study, we found that the expression of LINC00473 was elevated in Taxol-resistant CRC cells with decreased miR-15a levels. LINC00473 interacted with miR-15a and inhibited this tumor suppressor for the resistance to Taxol-induced cell apoptosis and tumor regression. However, Zhang et al. [18] found that ZBTB7A is an essential regulator in cisplatin-induced apoptosis via repressing the LINC00473–IL24 signaling axis for sustaining the osteosarcoma chemoresistance. These findings demonstrated the LINC00473, indeed, involved into the chemotherapy resistance, although the effects of LINC00473 had not been consistent in different tumors.

In conclusion, we here reported an lncRNA signature functioned as oncogene in CRC. Overexpressed LINC00473 predicted a poor clinical outcome for CRC patients and also promoted Taxol resistance and inhibited Taxol-induced tumor regression via regulating the tumor suppressor miR-15a. The mechanism involved with the inhibition of BCL-2-related anti-apoptosis pathway and up-regulated MDR signals.

## Abbreviations

CRC: colorectal cancer
DMEM: Dulbecco’s Modified Eagle’s medium
ILF2: interleukin enhancer binding factor 2
LncRNA: long non-coding RNA
MDR: multidrug-resistant
ncRNA: non-coding RNA
